# Integration of machine learning and bulk sequencing revealed exosome-related gene FOSB was involved in the progression of abdominal aortic aneurysm

**DOI:** 10.3389/fcell.2025.1554972

**Published:** 2025-05-22

**Authors:** Xianlu Ma, Hongjie Zhou, Ren Wang

**Affiliations:** ^1^ Shengli Clinical Medical College of Fujian Medical University, Fuzhou, China; ^2^ Department of Cardiac Vascular Surgery, Jining No.1 People’s Hospital, Jining, China; ^3^ Department of Cardiovascular Surgery, Fujian Provincial Hospital, Fuzhou, China

**Keywords:** abdominal aortic aneurysm, FOSB, exosome-related gene, inflammation, machine learning

## Abstract

**Background:**

Abdominal aortic aneurysm (AAA), characterized by the pathological dilation of the abdominal aorta, was associated with immune response and inflammation. However, the key genes involved in the occurrence and progression of AAA remains unclear.

**Methods:**

We applied Weighted Gene Co-expression Network Analysis (WGCNA) and Support Vector Machine Recursive Feature Elimination (SVM-RFE) to screen for significant genes from the Gene Expression Omnibus (GEO) dataset. The CIBERSORT algorithm was utilized to analyze the correlation between these genes and immune cell infiltration. Additionally, we validated the expression of FosB proto-oncogene, AP-1 transcription factor subunit (FOSB) in a murine model of AAA. FOSB was overexpressed and knocked out in vascular smooth muscle cells (VSMCs). Cell viability and apoptosis were assessed using the CCK-8 assay and flow cytometry, respectively. The levels of MMP2 and MMP9 in the cell supernatants were quantified by ELISA. The expression of contraction-related markers α-SMA and SM22α, and the synthetic marker OPN, was analyzed by qRT-PCR and Western blot.

**Results:**

A total of 44 differentially expressed genes were identified, revealing distinct expression patterns between AAA and normal samples. WGCNA identified two key gene modules that were strongly correlated with immune and inflammatory responses, with the hub genes from these modules enriched in immune-related pathways. FOSB was positively correlated with monocytes, plasma cells, eosinophils, and T follicular helper cells. It was further validated in an AngII-induced AAA mouse model. Overexpression of FOSB significantly increased the expression levels of MMP2 and MMP9 in VSMCs. Additionally, FOSB overexpression inhibited the expression of contractile phenotype markers α-SMA and SM22α, while promoting the expression of synthetic phenotype marker OPN.

**Conclusion:**

Exosome-related gene FOSB was involved in the progression of abdominal aortic aneurysm. FOSB represents a promising potential therapeutic target for mitigating the progression of Abdominal Aortic Aneurysm.

## Introduction

Abdominal aortic aneurysm (AAA) was an important public health disease, which was defined as the pathological and asymptomatic dilatation of the abdominal aorta (Golledge, 2019). Aortic rupture, a main complication, resulted in high mortality worldwide which was estimated at 0.15–0.2 million deaths approximately each year (GBD, 2013 Mortality and Causes of Death Collaborators, 2015). It was reported that risk factors, including increased age, gender, caucasian race, and heredity atherosclerotic risk factors, such as coronary artery disease and hypertension, contributed to the development and progression of AAA (Ullery et al., 2018). Furthermore, studies showed that inflammation was involved in the Pathophysiology of AAA. The aortic wall inflammation was a systemic process characterized by monocytes infiltration and cytokines production, accompanied by inflammatory factors secretion which included interleukins and tumor necrosis factor (TNF)-alpha (TNF-α), and the generation of reactive oxygen species, which both lead to the development of AAA (Miller et al., 2002). Moreover, it was reported that IgG, CD38, and GDF15 were potentially oxidative Stress and inflammatory markers in AAA (Sánchez-Infantes et al., 2021). Studies also showed that cathepsin, homocysteine, osteoprotegerin, and osteopontin were the most important biomarkers in AAA (Stepien et al., 2022). Therefore, it is essential to screen novel inflammatory biomarkers and effectors in the initiation and progression of AAA.

FOSB, also known as AP-1 transcription factor subunit, belonged to Fos subfamily and contained transcriptional activation domains (Shaulian and Karin, 2002), which played a central role in immune response, such as the activation of T cell and helper T cell differentiation (Atsaves et al., 2019). Moreover, it was reported that FOSB triggered to activate mitogen-activated protein kinase (MAPK) pathway and nuclear factor-κB (NF-κB) pathway to regulate proliferation, migration, and survival in cells and inflammation (Bhosale et al., 2022). It was reported that FOSB directly bound to the promoter regions of cytokines, such as IL-1, IL-6, and interacted with NF-κB to regulate their expression (Wang et al., 2019). FOSB regulated the expression of chemokines, such as monocyte chemoattractant protein-1 (MCP-1) and macrophage inflammatory protein-1α (MIP-1α), promoting the migration and accumulation of immune cells at the site of inflammation (Kaufmann et al., 2001). Furthermore, it was reported that c-Jun, another AP-1 transcription factor subunit, was upregulated in Angiotensin II (Ang II)-induced abdominal aortic aneurysm mouse model (Sun et al., 2019). Nevertheless, the precise molecular mechanism underlying the involvement of FOSB in the pathogenesis and progression of AAA is yet to be elucidated. In this study, we applied the Gene Expression Omnibus (GEO) dataset and performed differentially expressed genes analysis between AAA patients and healthy individuals. In addition, we performed Weighted Gene Co-expression Network Analysis (WGCNA) and Support Vector Machine Recursive Feature Elimination (SVM-RFE) to identify the potential hub genes in AAA. Furthermore, we constructed a protein and protein interaction (PPI) network and conducted functional enrichment analysis to identify molecular characteristics of hub genes. Overall, our study showed that FOSB was a potential biomarker and effector in the development of AAA.

## Materials and methods

### Data collection and processing

**FIGURE 1:**
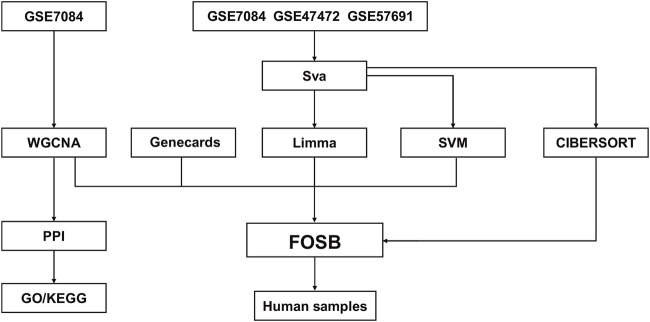
The overall workflow chart.

The workflow chart was presented in Figure 1. Gene expression profiles of datasets GSE47472 (including 14 AAA and 8 normal samples), GSE57691 (including 49 AAA and 10 normal samples), and GSE7084 (including 7 AAA and 8 normal samples) were downloaded from Gene Expression Omnibus (GEO). Data merging and batch effect correction were performed using the R package “sva” on three datasets and visualized by PCA analysis (Supplementary Figure S1). Differential gene expression analysis was conducted using the R package “limma”. The criterion for identifying differentially expressed genes (DEGs) was set as |logFC| > 1 and an adjusted *P* value < 0.05. The expression of DEGs was visualized using R package “ComplexHeatmap”. Exosome-related genes were acquired from Genecards.

### Weighted gene co-expression network analysis

WGCNA was applied to screen co-expression gene modules and significant genes associated with the occurrence of AAA (Langfelder and Horvath, 2008). GSE7084 was used to perform WGCNA using R package “WGCNA”. Hierarchical clustering and dynamic tree cutting were used to screen modules of genes that are highly co-expressed. The correlation between gene modules and the occurrence of AAA was analyzed based on Pearson correlation analysis. GS (gene significance) and MM (module membership) were two important measures used to identify biologically meaningful gene modules, where GS denoted the correlation between the expression of each gene and the occurrence of AAA and MM represented the correlation between the expression of each gene and the given module. The correlation between a gene’s GS and its MM has a *P*-value less than 0.05, it suggests that the gene is highly connected within its corresponding module and strongly associated with the phenotype of interest.

### Construction of protein and protein interaction network

The candidate hub gene modules were extracted and utilized to construct a PPI network using The Search Tool for The Retrieval of Interaction Genes (STRING). The hub genes were overlapped and identified based on the five methods in CytoHubba, a plugin of Cytoscape. The five methods were MNC (maximal neighborhood component), MCC (maximal clique centrality), EPC (edge percolated component), degree (node connect degree), and closeness (node connect closeness), respectively.

### Functional enrichment analysis

The overlapped hub genes were applied to perform Gene Ontology (GO) and Kyoto Encyclopedia of Genes and Genomes (KEGG) enrichment analyses using online database (The Database for Annotation, Visualization, and Integrated Discovery (DAVID)). The cutoff of the criterion was set as *P* < 0.05. The visualization of enrichment analysis using R package “ggplot2”.

### Support vector machine recursive feature elimination

SVM-RFE was a machine learning algorithm to select the most relevant gene features associated with the occurrence of AAA (Sanz et al., 2018). The merged dataset was divided into a training and test dataset. The SVM model was trained using four kernels, including linear, polynomial, radial, and sigmoid, and subjected to 10-fold cross-validation to improve the accuracy and efficiency. Then, receiver operating characteristic (ROC) curves were plotted for each gene feature using the R package “pROC” to evaluate the performance of the model. The final intersected genes were visualized using R package “VennDiagram”.

### Immune infiltration analysis

The immune infiltration within the merged dataset was analyzed using CIBERSORT algorithm. The correlation between immune infiltration and the expression of FOSB was analyzed using Pearson correlation analysis. The cutoff of the criterion was set as *P*-value < 0.05.

### Animal model of AAA and tissue collection

Animal experiments were performed in strict accordance with the guidelines of the Ethics Committee of Fujian Medical University (approval number: 2023-07-19A). Eight-week–old male C57/BL6J mice were purchased from laboratory animal center of Fujian Medical University. The mice were anesthetized with 2% isoflurane inhalation. Then, a small incision was made on the skin behind the scapula, and the Alzet osmotic pump (Model 2004) was gently placed through the incision and positioned behind the scapula. The mice were randomly divided into AAA and normal groups. AAA group (n = 6) was subcutaneously infused with Angiotensin II (AngII, 1 μg/kg/min), and the normal group (n = 6) was subcutaneously infused with 0.9% (v/v) saline. Finally, the skin incision was sutured using 6–0 sutures. The pump was applied to deliver AngII subcutaneously for 3 weeks.

The abdominal aorta was extracted after 3 weeks. In anesthetized adult mice, lung tissues were extracted. Subsequently, the abdominal aorta was transected, and ice-cold phosphate-buffered saline (PBS) was used to perfuse the abdominal aorta. Then, the abdominal aorta was dissected under a microscope (Leica, Germany). Tissues were rapidly frozen in liquid nitrogen and maintained at −80°C for further experiments.

### Ultrasonography of the mouse abdominal aorta

The upper abdominal region of the mice was depilated using a laboratory animal hair remover. The animals were then placed in a gas anesthesia machine. After successful anesthesia, the mice were positioned in the supine position on the surgical platform. Ultrasound gel was applied to the transducer, while continuous gas anesthesia was maintained via a nasal cannula. The limbs of the mice were secured to electrode pads. The transverse section of the abdominal aorta was visualized in the upper abdominal region, and detailed imaging data were acquired. The maximum diameter of the abdominal aorta was measured using Doppler ultrasound, with three independent measurements recorded for accuracy. Mice were imaged using the VEVO 2100 ultrasound system (FUJIFILM VisualSonics, Canada).

### Cell culture

Vascular smooth muscle cells (VSMCs) were cultured in DMEM supplemented with 10% FBS and 1% P/S at 37°C in a 5% CO2 incubator with 95% relative humidity. When the cell density reached 80%, the cells were digested with 1 mL of 0.25% trypsin for 1 min. Digestion was terminated by adding 2 mL of complete medium, followed by subculturing. The experiment consisted of three groups: control, oe-FOSB (FOSB overexpression), and si-FOSB (FOSB knockdown). All groups were treated with PDGF (platelet-derived growth factor, 10 ng/μL) for 24 h to induce the dedifferentiation of VSMCs, simulating the inflammatory environment during the onset of AAA *in vivo*.

### FOSB overexpression vector construction

The pcDNA3.1 vector (synthesized by Shanghai Shenggong Biotech) was used to construct the pcDNA3.1-FOSB plasmid based on the FOSB sequence obtained from NCBI (Supplementary Table S1). The plasmid was transfected into cells, which were cultured in 6-well plates to approximately 80% confluence to ensure healthy cell status. 125 μL of diluted Lipofectamine 3,000 reagent was mixed with 125 μL of diluted plasmid and incubated at room temperature for 15 min, followed by transfection into VSMCs in the 6-well plate. The overexpression of FOSB in the cells was confirmed by RT-qPCR.

### FOSB knockdown vector construction

siRNA sequences targeting the FOSB gene CDS (NM_001347586.1:615-1328) were designed (Supplementary Table S2). 2 μL of 20 μM siRNA duplex was added to 200 μL of serum-free medium and gently pipetted to mix. Then, 10 μL of RNAFit transfection reagent (HanHeng Biotech, HB-RF-1000) was added, and the mixture was vortexed for 10 s and incubated at room temperature for 10 min to form the transfection complexes. The original medium was replaced with 1.8 mL of pre-warmed complete medium with serum, and the transfection complex was added to the cells. The cells were gently shaken to mix. The final volume per well was 2 mL, and the siRNA concentration was 20 nM. After 48 h of incubation, the cells were analyzed.

### Cell viability assay

Logarithmic phase cells were seeded at 5,000 cells/well in 96-well plates and incubated at 37°C in a 5% CO_2_ incubator for 6 h. Each group consisted of six replicates. After 24 h of incubation, 10 μL of CCK-8 solution was added to each well 2 h before the end of incubation. The absorbance was measured at 450 nm using a microplate reader.

### Flow cytometry

Logarithmic phase cells were seeded at 1 × 106 cells/well in 6-well plates and cultured for 24 h at 37°C in a 5% CO2 incubator. Cells were collected by centrifugation at 1000 rpm for 5 min, and the supernatant was discarded. The cells were resuspended in 1 mL PBS and washed once. A pre-prepared 1X Annexin V Binding Solution was added, and the cell concentration was adjusted to 1 × 106 cells/mL. 100 μL of the cell suspension was mixed with 5 μL of Annexin V-FITC and 5 μL of PI solution and incubated at room temperature in the dark for 15 min 400 μL of 1X Annexin V Binding Solution was then added to the cells, which were mixed gently and analyzed by flow cytometry within 1 h.

### Enzyme-linked immunosorbent assay

Logarithmic phase cells (1 × 10^6^ cells/well) were seeded in 6-well plates and cultured for 24 h at 37°C in a 5% CO_2_ incubator. The supernatant was collected by centrifugation at 1,000 ×g for 20 min and analyzed by ELISA. Standards and samples (50 μL) were added to the wells of the enzyme-coated plate, which was then sealed and incubated at 37°C for 30 min. After incubation, the plate was washed five times with washing buffer, and 50 μL of enzyme-labeled reagent was added to each well. The plate was incubated at 37°C for 30 min, followed by washing again. Then, 50 μL of substrate A and B were added to each well, and the plate was incubated at 37°C for 15 min in the dark. The reaction was stopped with 50 μL of stop solution, and the absorbance (OD value) at 450 nm was measured.

### Quantitative real-time polymerase chain reaction

The abdominal aorta tissue was homogenized, and total RNA was extracted using the TRIzol reagent following the manufacturer’s instructions. The synthesized cDNA was obtained using the HiScript lll 1st Strand cDNA Synthesis Kit. (Vazyme, China). Then, gene expression was performed using Taq Pro Universal SYBR qPCR Master Mix (Vazyme, China) with CFX96 Touch 1855195 Real-time PCR instrument (Bio-Rad, United States). Glyceraldehyde-3-phosphate dehydrogenase (GAPDH) was used as the internal control. The final relative expression was calculated using the 2^−ΔΔCq^ method (Livak and Schmittgen, 2001). The primers are shown in Supplementary Table S3. The experiments were performed as three independent biological replicates, using three technical repeats for each biological repeat.

### Western blot

The abdominal aorta was homogenized and lysed with cold RIPA containing 1% PMSF. The whole tissue lysates were centrifuged at 12,000 × g for 20 min. The supernatant was transferred to a new microtube kept on ice, and the protein concentration was determined using the Pierce BCA Protein Assay Kit (Thermo Fisher Scientific, United States) according to the manufacturer’s instructions. Subsequently, 15 μg of the protein was separated on a 10% Sodium Dodecyl Sulfate Polyacrylamide Gel Electrophoresis (SDS-PAGE) and transferred to a polyvinylidene fluoride (PVDF) membrane. The PVDF membrane was blocked using 5% defatted milk at room temperature for 2 h and incubated for four primary antibodies against CCR7 (1:5,000, Abcam, United Kingdom), FOSB (1:5,000, Abcam, United Kingdom), IL1B (1:5,000, Abcam, United Kingdom), MMP9 (1:5,000, Abcam, United Kingdom),α-SMA (1:1000, Abcam, United Kingdom), SM22α (1:1000, Abcam, United Kingdom), OPN (1:1000, Abcam, United Kingdom), GAPDH (1:10,000, Bioss, China) at 4°C overnight. The membrane was washed three times using TBST and then incubated for corresponding horseradish peroxidase (HRP)-conjugated anti-rabbit IgG secondary antibodies (1:10,000) at room temperature for 1 h. The intensity of the bands was visualized using JP-K6000 chemiluminescence detection system and analyzed using ImageJ software.

### Statistical analysis

Unpaired *t*-tests were employed to compare continuous variables between the two groups. The bioinformatical statistical analyses were conducted using the R software (version 4.2.1) and the results of qRT-PCT and Western blot were analyzed and visualized using GraphPad Prism 9 (version 9.4.0). The *p-*value < 0.05 was considered statistically significant.

## Results

### Identification of DEGs in AAA

**FIGURE 2:**
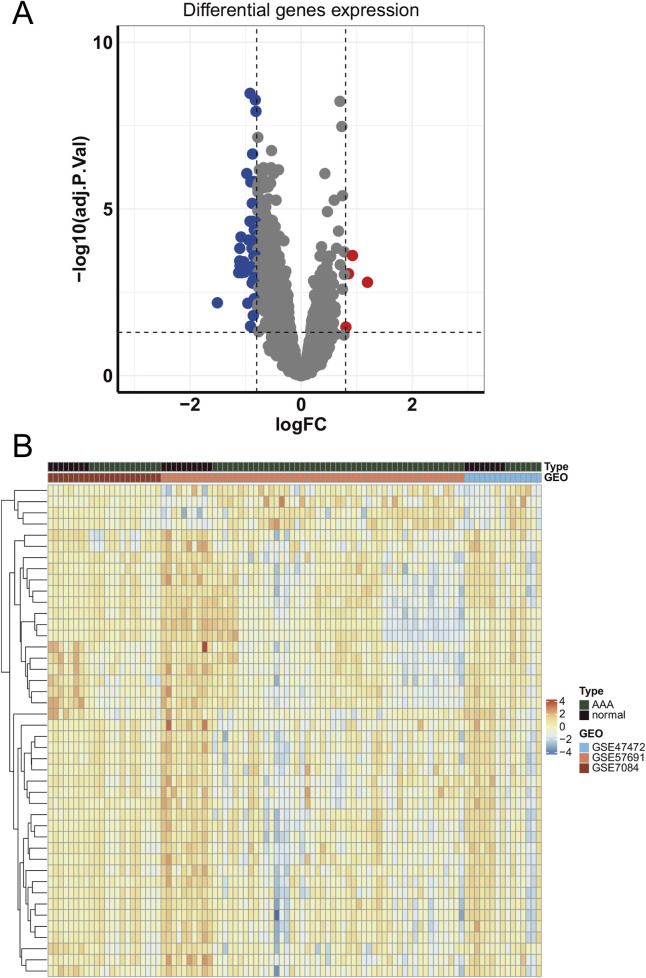
The expression of differentially expressed genes in GEO datasets. **(A)** The volcano plot of differentially expressed genes in merged GEO datasets. **(B)** The heatmap of differentially expressed genes.

To identify differentially expressed genes, we merged three GEO datasets (GSE47472, GSE57691, and GSE7084) and conducted differential genes analysis. Results showed that 44 DEGs were identified, including 40 downregulated genes and 4 upregulated genes (Figure 2A). Moreover, the expression of DEGs had a distinct pattern between AAA and normal samples in merged GEO datasets (Figure 2B).

### Identification of the hub gene modules in the occurrence of AAA

**FIGURE 3:**
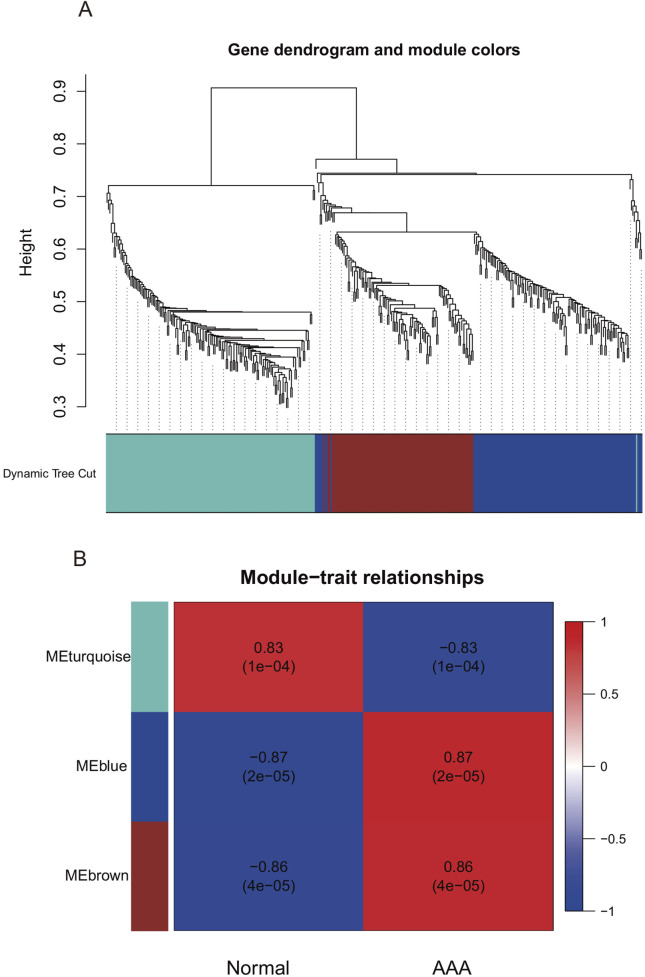
Weighted Gene Co-expression Network Analysis in GSE7084. **(A)** Gene dendrogram of GSE7084. **(B)** The correlation between gene modules and clinical traits.

**FIGURE 4:**
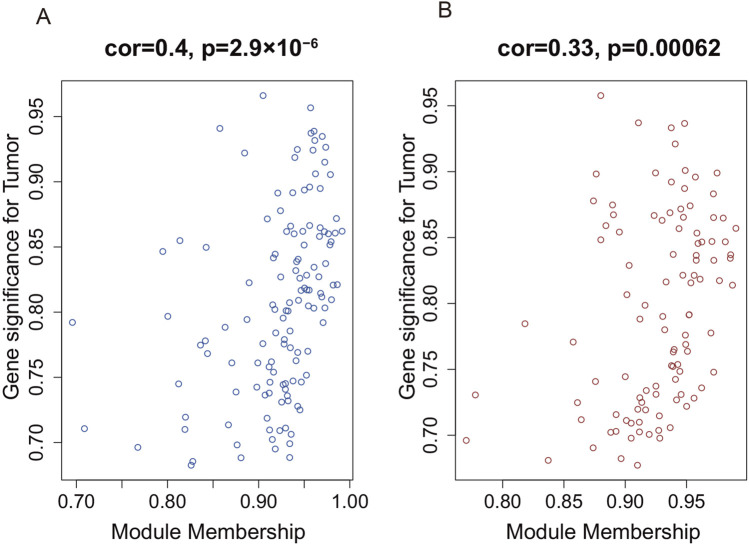
The correlation between gene significance and module membership in WGCNA. **(A)** The correlation between gene significance and module membership in the blue module. **(B)** The correlation between gene significance and module membership in the brown module.

To identify the hub gene modules associated with the occurrence of AAA, we performed WGCNA using the GSE7084 dataset, including 7 AAA and 8 normal samples, and 382 DEGs in GSE7083 were applied to construct the co-expression network. The result showed that 382 DEGs were clustered into three gene modules, which were turquoise, blue, and brown gene modules, respectively (Figure 3A). To investigate the correlation between gene modules and the occurrence of AAA, we conducted a Pearson correlation analysis. The result showed that blue and brown gene modules were strongly correlated with the occurrence of AAA (Cor = 0.87, p-value = 2 × 10^−5^, and Cor = 0.86, p-value = 4 × 10^−5^) (Figure 3B). Furthermore, we explored the correlation between GS and MM. The result showed that GS had a strong correlation with MM in the blue module (Cor = 0.4 and p-value = 2.9 × 10^−6^) and brown module (Cor = 0.33 and p-value = 0.00062) (Figures 4A,B), which indicated that genes in the blue and brown module were strongly associated with AAA. Genes in blue and brown gene modules were extracted for further analysis.

### Hub genes were associated with immune response and inflammatory response

**FIGURE 5:**
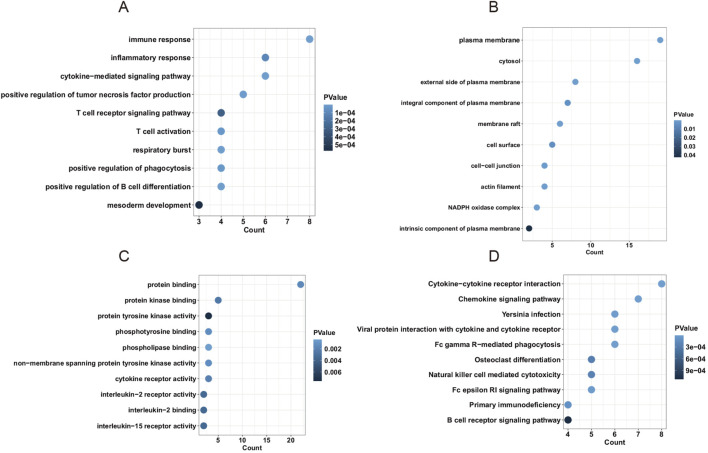
The enrichment analysis of hub genes in the PPI network. **(A)** The biological process (BP) term of hub genes. **(B)** The cellular component (CC) term of hub genes. **(C)** The molecular function (MF) term of hub genes. **(D)** The KEGG pathway of hub genes.

To identify candidate hub genes, a protein-protein interaction network was constructed and analyzed using Cytoscape. The result showed that 24 overlapped genes were screened based on five plugin methods (MNC, MCC, EPC, Degree, and Closeness) of Cytoscape (Supplementary Table S4). Then, overlapped genes were applied to perform enrichment functional analysis. To investigate the biological function and molecular pathway of hub genes, we performed the enrichment functional analysis. Results showed that genes were enriched in immune response, inflammatory response, and cytokine-mediated signaling pathway in biological process (BP) term (Figure 5A). Moreover, genes were highly enriched in the plasma membrane, cytosol, and external side of the plasma membrane in cellular component (CC) term and protein binding, protein kinase binding, and protein tyrosine kinase activity in molecular function (MF) term (Figures 5B,C). Furthermore, genes were highly enriched in cytokine-cytokine receptor, chemokine signaling pathway, and *Yersinia* infection in the KEGG pathway (Figure 5D). Taken together, hub genes in blue and brown modules were strongly associated with immune and inflammatory responses in the progression of AAA.

### FOSB was associated with the occurrence of AAA

**FIGURE 6:**
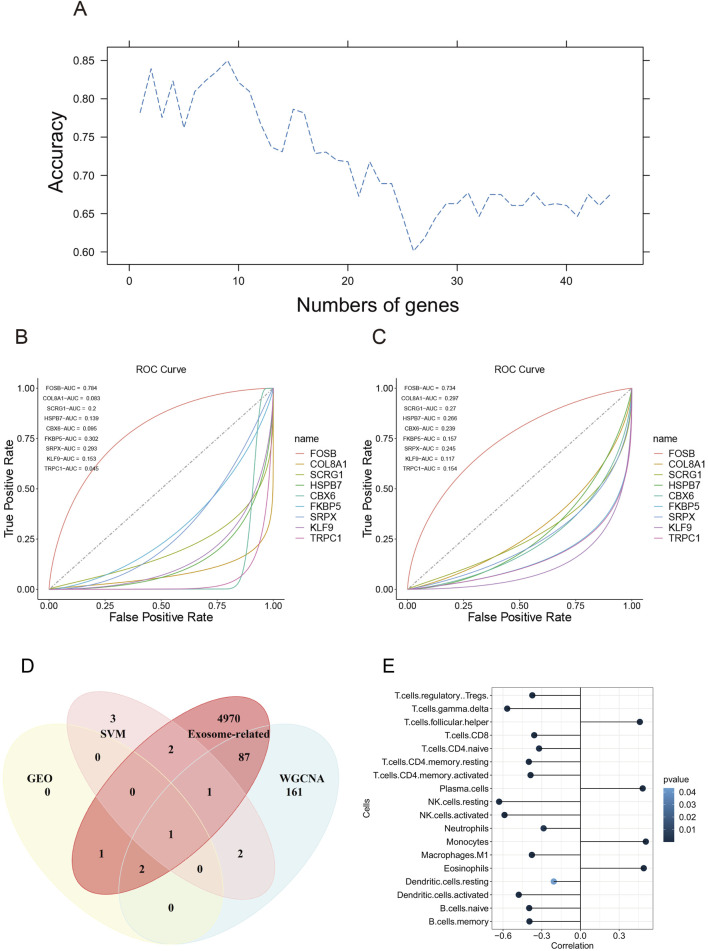
Gene features selection using support vector machine recursive feature elimination. **(A)** The accuracy of the number of gene features using support vector machine algorithm. **(B)** The ROC curve of 9 gene features in the test dataset. **(C)** The ROC curve of 9 gene features in the training dataset. **(D)** The Venn diagram of genes screened in GEO datasets, SVM, exosome-related genes, and WGCNA. **(E)** The correlation between FOSB and immune infiltration.

To screen significant gene features, we performed support vector machine recursive feature elimination. We divided the merged dataset into a training and test dataset at the proportion of 7:3 and applied four kernels of the support vector machine algorithm. The result showed that the linear kernel had the best performance on the classification of AAA (Data not shown). Then, we applied 10-fold cross-validation to improve accuracy and select significant gene features. The result showed that 9 gene features had the most performance of the SVM model, which were FOSB, COL8A1, SCRG1, HSPB7, CBX6, FKBP5, SRPX, KLF9, and TRPC1 (Figure 6A).

To evaluate the performance of the classification model, we plotted the ROC curve of each gene. The results showed that FOSB had the best performance with AUC being 0.784 in the test dataset and 0.734 in the training dataset, whereas other genes except FOSB had a bad performance in the training and test dataset (Figures 6B,C). Then. We downloaded exosome-related genes in Genecards online database and intersected them with genes screened by DEGs in GEO, SVM, and WGNCA. Finally, we found that FOSB was the sole gene associated with the occurrence of AAA (Figure 6D), indicating that FOSB may be involved in the development of AAA.

### FOSB was associated with immune infiltration in the occurrence of AAA

To investigate the potential correlation between FOSB and immune infiltration in AAA, we utilized the CIBERSORT algorithm to perform immune cell infiltration analysis. The result showed that FOSB was strongly correlated with 18 immune infiltrating cells (Figure 6E). Moreover, the expression of FOSB was strongly positively correlated with plasma cells, monocytes, eosinophils, and T follicular helper cells and negatively correlated with most T cells, including CD4^+^ and CD8^+^ T cells, and B cells, natural killer cells, and dendritic cells.

### The expression of four DEGs was validated using the AAA animal model

**FIGURE 7:**
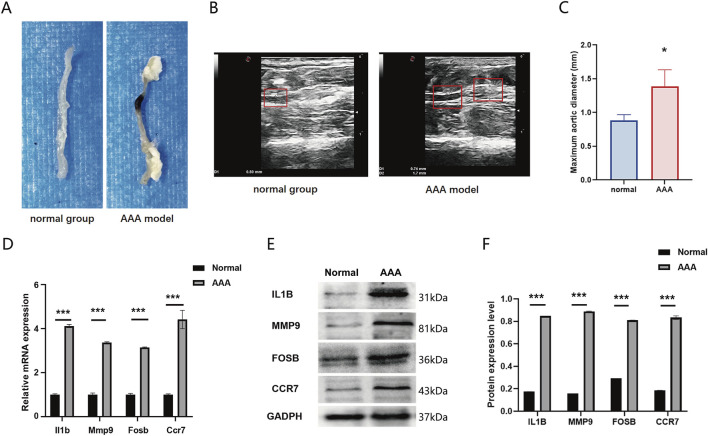
**(A)** The representative photographs illustrate the macroscopic features of the aortas in the AAA group and the normal group. **(B)** Representative ultrasonographic images of the two groups of mice **(C)** Comparison of the maximum abdominal aortic diameters between the two groupsThe expression of FOSB and differentially expressed genes in AAA mice model. **(D)** The mRNA expression of Fosb, Il1b, Mmp9, and Ccr7 in AAA and normal group. **(E, F)** The protein expression of FOSB, IL1B, MMP9, and CCR7 in AAA and normal group. n = 6. *P < 0.05, **P < 0.01, ***P < 0.001.

The representative photographs illustrate the macroscopic features of the aortas in the AAA group and the normal group. Compared with the control group, the abdominal aortic wall of the model group exhibited expansive bulging, aneurysmal dilation, and a significantly increased diameter (Figure 7A). Ultrasonographic results of the abdominal aorta revealed that all mice in the AAA group demonstrated a larger aortic diameter compared to the Normal group (*P* = 0.028, Figures 7B,C). To validate the expression of DEGs in GEO datasets, we constructed an AngII-induced AAA mouse model and extracted the abdominal aorta to examine the mRNA and protein expression. Results showed that the mRNA expression of Fosb, Ccr7, Il1b, and Mmp4 was upregulated in the AAA group compared with that in the normal group (Figure 7D). Consistently, the protein expression was also upregulated in the AAA group. Therefore, our results validated the expression of FOSB, CCR7, IL1B, and MMP9 in AAA mice model and indicated that these four genes were involved in the occurrence and progression of AAA (Figures 7E,F).

### FOSB inhibits proliferation and promotes apoptosis in dedifferentiated VSMCs

**FIGURE 8:**
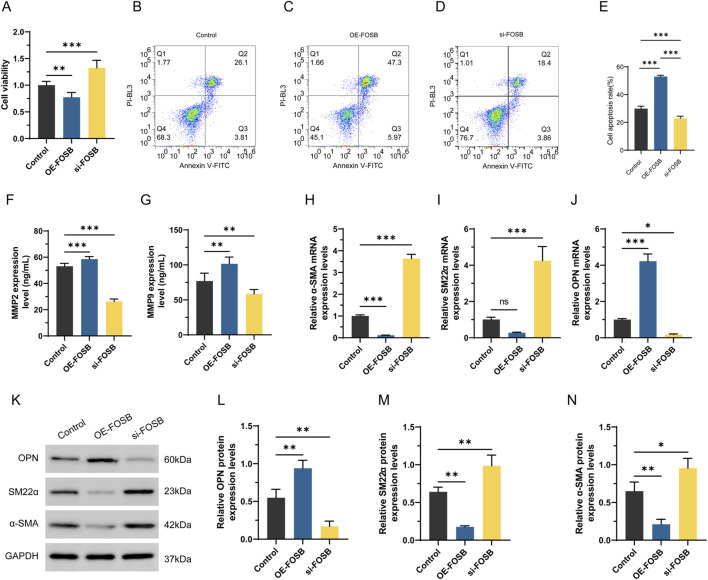
The effect of FOSB on proliferation, apoptosis, and phenotypic transition markers in differentiated VSMCs. **(A)** Detection of CCK8 cell activity. **(B–E)** Apoptosis was detected by flow cytometry. **(F, G)** The levels of MMP2 and MMP9 in the cell supernatant were detected by ELISA. **(H–J)** Expression of α-SMA, SM22αand OPN detected by RT-PCR. **(K–N)** Western blot analysis of α-SMA and SM22α OPN protein expression. n = 6. *P < 0.05, **P < 0.01, ***P < 0.001.

The effect of FOSB on the viability and migratory capacity of dedifferentiated VSMCs was assessed using the CCK-8 assay to explore its potential regulatory role in the pathogenesis of AAA (Figure 8A). The results showed that after 48 h of PDGF treatment, interference with FOSB expression significantly enhanced cell viability, with a marked difference compared to the negative control group (*P* < 0.01). In contrast, FOSB overexpression suppressed VSMC viability, and this difference was also statistically significant compared to the control group (*P* < 0.05). Furthermore, apoptosis in the oe-FOSB and si-FOSB groups was analyzed by flow cytometry (Figures 8B–E). After 48 h of PDGF treatment, the apoptosis rate was significantly increased in the oe-FOSB group compared to the control group, whereas the apoptosis rate in the si-FOSB group was significantly decreased (*P* < 0.01).

### FOSB modulates the expression of contractile and synthetic phenotype markers in VSMCs in AAA

To investigate the effect of FOSB on the expression of MMP2 and MMP9, the levels of these proteins in the culture supernatant were measured. The results showed that, compared to the control group, the expression levels of MMP2 and MMP9 were significantly higher in the OE-FOSB group, while they were significantly reduced in the si-FOSB group, with statistically significant differences (*P* < 0.01, Figures 8F,G). These findings suggested that FOSB might promote the formation of AAA by upregulating the expression of MMP2 and MMP9. Moreover, qRT-PCR results showed that the expression of α-SMA and SM22α was significantly decreased in the OE-FOSB group, while the expression of OPN was significantly increased (*P* < 0.05). Conversely, the si-FOSB group exhibited the opposite trend (*P* < 0.05, Figures 8H–J). Consistent results were obtained from Western blot analysis, further confirming the regulatory effect of FOSB on these proteins (*P* < 0.05, Figures 8K–N).

## Discussion

In this study, we identified FOSB was regulated in AAA samples in the merged GEO dataset and found that FOSB was selected as a potential hub gene using different bioinformatical methods, including WGCNA and SVM-RFE. In the meantime, we found that FOSB was an exosome-related gene in the Genecards database. In addition, we found that other genes involved in the occurrence of AAA in WGCNA were highly enriched in immune and inflammation response. We also found that FOSB had an excellent performance for classifying AAA and normal samples. Furthermore, we found that FOSB was strongly correlated with immune infiltration, including plasma cells, monocytes, eosinophils, and T follicular helper cells. FOSB expression in vascular smooth muscle cells (VSMCs) influences their phenotypic transformation, promoting MMP2/MMP9 expression, potentially accelerating abdominal aortic aneurysm (AAA) development. Therefore, our study indicated that FOSB might be a potential biomarker and therapeutic target in the occurrence and progression of AAA via regulating immune and inflammation responses.

In differentially expressed genes analysis, we found that 4 DEGs were upregulated in AAA samples, which were CCR7, IL1B, MMP9, and FOSB, and validated the expression of these four genes in the AAA mice model. CC chemokine receptor 7 (CCR7) was a G protein-coupled receptor (GPCR), which mediated the Dendritic cells (DCs) migration and guided matured DCs into lymph nodes (Rodríguez-Fernández and Criado-García, 2020). CCR7 and its ligands, CCL19 and CCL21, were crucial signaling axis to regulate immune cell traffic (Hong et al., 2022). It was reported that the expression of CCR7 was upregulated in AAA patients using the immunohistochemical staining method (Wan et al., 2018). However, it was novel that notch inhibition recruited CCR7-positive DCs to induce the regression of active AAA (Hans et al., 2020), which indicated that CCR7 might have a beneficial effect on the development of AAA. IL1B, as a pro-inflammatory factor, was activated by NLR family pyrin domain containing 3 (NLRP3) inflammasome activation and NF-κB transcription, which was implicated in AAA (Shi et al., 2021). Studies also showed that the expression of IL-1β was higher in aneurysmal patients than that in non-aneurysmal patients (Shi et al., 2021). Matrix metalloproteinase 9 (MMP9) was a crucial proteolytic enzyme, characterized by degrading extracellular components in the aortic wall (Howatt et al., 2017). It was reported that the expression of MMP9 was upregulated and lymphocytes in human AAA tissue had a higher positive rate of MMP9 staining (Li et al., 2021). Overall, the results of upregulated DEGs in our research were consistent with those in previous studies.

FOSB was a subunit of the AP-1 transcription factor that was involved in the inflammation response. Most studies reported that FOSB was associated with vascular inflammation, The expression of FOSB was suppressed by dibenzoxazepinone BT2 which inhibited angiogenesis and vascular permeability (Li et al., 2020a). The expression of FOSB was also upregulated under the knockdown of Jumonji domain-containing protein 1 A, which increased inflammatory response and oxidative stress in human umbilical vein endothelial cells (Zhao et al., 2021). Furthermore, downregulated FOSB and MMP9 were found under the treatment of Insulin-like growth factor 1 that reduced reduces coronary atherosclerosis (Sukhanov et al., 2023). In our study, we found that the expression of FOSB was upregulated in the merged GEO dataset and had an excellent performance on the classification of AAA and normal individuals. Intriguingly, FOSB was identified as a novel validated gene in the pathobiology of AAA in “AAA-chip”, which was in accordance with our study (Hinterseher et al., 2013).

Inflammation was associated with the progression of AAA, which was characterized by the infiltration of inflammatory cells into the aortic wall, leading to the breakdown of collagen and elastin (Anagnostakos and Lal, 2021). Various immune cells were activated in chronic inflammation, evoking the progression of AAA (Skotsimara et al., 2022). In our study, we found that AAA-related genes in WGCNA gene modules were highly enriched in immune response and inflammatory response in BP terms and in cytokine-cytokine receptor interaction and Chemokine signaling pathway in the KEGG pathway, which was consistent with the previous studies. Furthermore, we showed that the expression of FOSB was positively correlated with monocytes, plasma cells, eosinophils, and T follicular helper cells. The recruitment of monocyte to the aortic wall was involved in CCL2 and CCR2, a chemokine-chemokine receptor pathway. CCL2 and IL6 induced the recruitment of monocyte into AAA wall, which was then differentiated into macrophages and promoted the proliferation of adventitial fibroblast (Márquez-Sánchez and Koltsova, 2022). Moreover, knock-out CD11b, a monocyte integrin subunit, decreased the expression of MMP9 and IL6 and attenuated the elastin and collagen degradation (Raffort et al., 2017). T follicular helper cells (Tfh) were implicated to the regulation of autoimmune and inflammatory responses. It was reported that Tfh cells were in the aorta wall and increased in atherogenic environment (Zhou et al., 2021). Eosinophils were important innate immune cells that regulated macrophage phagocytosis and smooth muscle cell contraction. A recent study had shown that eosinophils protected mice from AAA growth by releasing IL4 and eosinophils-associated-ribonuclease-1 to regulate the polarization of macrophage and monocyte (Baptista et al., 2018).

Matrix metalloproteinases (MMPs) were primarily responsible for the degradation of extracellular matrix (ECM) *in vivo*, with MMP-2 and MMP-9 being key markers of AAA (Li et al., 2020b). The phenotypic transformation of VSMCs and the resulting imbalance in ECM degradation is one of the critical mechanisms underlying AAA formation (Shapouri-Moghaddam et al., 2019). VSMC phenotypes were classified into contractile and synthetic types, with synthetic VSMCs having stronger secretory functions and being one of the main sources of MMPs (Tompkins et al., 2015). Our results indicated that changes in FOSB expression in VSMCs can influence cellular biological behaviors, including promoting cell dedifferentiation, enhancing MMP2/MMP9 expression, reducing the expression of contractile phenotype markers, and inducing apoptosis. These effects might lead to VSMC dysfunction, thereby accelerating the development of AAA. Furthermore, the regulation of FOSB may affect arterial wall stability by modulating apoptosis and affecting extracellular matrix remodeling. Therefore, FOSB could be a potential therapeutic target for AAA. However, future research is required to further explore the specific molecular mechanisms of FOSB and evaluate its feasibility as a clinical therapeutic target.

Exosome was a type of membrane-bound extracellular vesicles (EVs) containing bioactive molecules, playing an essential role in cellular communications (Liu et al., 2021). Exosomes derived from various cell types could induce inflammasome activation and IL-1β secretion, such as chondrocytes and Bone marrow-derived macrophages (Liu et al., 2021). Although there was less evidence that proved the relationship between exosome and FOSB, the association between exosome and AP-1 had been discovered. It was reported that M2 macrophage-derived exosomes promoted the dedifferentiation of vascular smooth muscle cells (VSMCs) by the activation of c-Jun/AP-1 pathway (Liu et al., 2024). Furthermore, exosomes were detected in the adventitia of AAA, which contained a higher accumulation of macrophage (Vandestienne et al., 2021). Inhibition of macrophage-derived exosomes decreased the expression of MMP2 and attenuated the progression of AAA (Li et al., 2020b). In our study, we showed that FOSB was an exosome-related gene using bioinformatical method and validated that the expression of FOSB was upregulated in the AAA mouse model.

In this study, overexpression of FOSB significantly increased the expression levels of MMP2 and MMP9 in VSMCs. It was demonstrated that FOSB binds to MMP-2 and MMP-9 promoters via AP-1 complex activation, thereby inducing their overexpression in VSMCs and promoting matrix degradation (Shapouri-Moghaddam et al., 2019). This promoted the sorting of these proteases, along with inflammatory factors such as IL-1β, into exosomes. Exosomes carrying MMPs target vascular wall fibroblasts and endothelial cells through intercellular communication, where they release active MMPs into the local microenvironment (Tompkins et al., 2015; Hummers et al., 2009). These enzymes specifically cleave elastin and type IV collagen, disrupting the structure of the aortic media (Li et al., 2018; Binder et al., 2015). Additionally, FOSB causes a dysregulation in the ratio of precursors of MMPs and tissue inhibitors of metalloproteinases via exosome-mediated pathways, creating a sustained proteolytic microenvironment (Wang et al., 2014). Ultimately, this cascade leads to the loss of tensile strength in the vessel wall, regional dilation, and irreversible aneurysm progression.

This study has several limitations. First, our study primarily relied on an AngII-induced AAA mouse model and *in vitro* experiments using VSMCs. Validation in human tissues or primary human cells would significantly strengthen the conclusions. Second, the functional association between FOSB and exosomes has not been directly validated experimentally limited by the accessibility of clinical exosome samples. Although it was demonstrated that FOSB regulates MMP2/MMP9 expression and VSMC phenotype switching markers, the precise molecular mechanisms underlying these effects remain unclear. Future studies should collaborate with clinical centers to establish an exosome sample repository from aneurysm patients to further advance the research. In this study, we identified FOSB as a key gene involved in the pathogenesis of AAA, particularly in the regulation of immune responses, inflammation, and ECM remodeling. Although FOSB may play a significant role in these interconnected processes, its specific interactions remain insufficiently explored. It is currently unclear whether FOSB directly influences immune cell behavior and drives the inflammatory response in the aortic wall. Additionally, the potential activation of matrix metalloproteinases MMPs by inflammation remains to be fully elucidated, as these enzymes degrade ECM components like collagen and elastin, leading to structural instability of the aortic wall. By linking immune activation with ECM degradation, FOSB appears to play a pivotal role in the inflammatory and structural changes that accelerate AAA progression. However, the precise biological mechanisms underlying FOSB’s involvement in these processes warrant further investigation.

## Conclusion

In this study, we found that FOSB, an exosome-related gene, was involved in the occurrence and progression of AAA using bioinformatical method and machine learning, and validated the expression of FOSB, CCR7, IL1B, and MMP9 in AAA mice model. Furthermore, we showed that the expression of FOSB was positively correlated with monocytes, plasma cells, eosinophils, and T follicular helper cells, and AAA-related genes in WGCNA module were highly enriched in immune and inflammation response. Overall, our results indicated that AAA was an immune and inflammation-related disease using bioinformatical analysis and validated that FOSB was involved in the progression of AAA.

## Data Availability

The original contributions presented in the study are included in the article/Supplementary Material, further inquiries can be directed to the corresponding author.
