# Supplementary material for: Integration of machine learning and bulk sequencing revealed exosome-related gene FOSB was involved in the progression of abdominal aortic aneurysm
Source: Front Cell Dev Biol. 2025 May 22;13:1554972. doi: 10.3389/fcell.2025.1554972 (PMC12142044; doi:10.3389/fcell.2025.1554972)

Table S1. The sequence of FOSB

| The sequence of FOSB |
| --- |
| ATGTTTCAAGCTTTTCCCGGAGACTACGACTCCGGCTCCCGGTGTAGCTCATCACCCTCCGCCGAGTCTCAGTACCTGTCTTCGGTGGACTCCTTCGGCAGTCCACCCACCGCCGCCGCCTCCCAGGAGTGCGCCGGTCTCGGGGAAATGCCCGGCTCCTTCGTGCCAACGGTCACCGCAATCACAACCAGCCAGGATCTTCAGTGGCTCGTGCAACCCACCCTCATCTCTTCCATGGCCCAGTCCCAGGGGCAGCCACTGGCCTCCCAGCCTCCAGCTGTTGACCCTTATGACATGCCAGGAACCAGCTACTCAACCCCAGGCCTGAGTGCCTACAGCACTGGCGGGGCAAGCGGAAGTGGTGGGCCTTCAACCAGCACAACCACCAGTGGACCTGTGTCTGCCCGTCCAGCCAGAGCCAGGCCTAGAAGACCCCGAGAAGAGACACTTACCCCAGAAGAAGAAGAAAAGCGAAGGGTTCGCAGAGAGCGGAACAAGCTGGCTGCAGCTAAGTGCAGGAACCGTCGGAGGGAGCTGACAGATCGACTTCAGGCGGAAACTGATCAGCTTGAAGAGGAAAAGGCAGAGCTGGAGTCGGAGATCGCCGAGCTGCAAAAAGAGAAGGAACGCCTGGAGTTTGTCCTGGTGGCCCACAAACCGGGCTGCAAGATCCCCTACGAAGAGGGGCCGGGGCCAGGCCCGCTGGCCGAGTGA |

Table S2. The sequence of siRNA FOSB

| siRNA | sequence |
| --- | --- |
| FOSB siRNA-1 | 5’-AAGAGAUGAGGGUGGGUUGCA-3’  3’-CAACCCACCCUCAUCUCUUCC-5 |
| FOSB siRNA-2 | 5’-UCUUGCAGCCCGGUUUGUGGG-3’  3’-CACAAACCGGGCUGCAAGAUC-5’ |
| FOSB siRNA-3 | 5’-UUCGUAGGGGAUCUUGCAGCC-3’  3’-CUGCAAGAUCCCCUACGAAGA-5’ |
| si-NC | 5'-UUCUCCGAACGUGUCACGUTT-3'  5'-ACGUGACACGUUCGGAGAATT-3' |

Table S3. The primer of genes

| Gene | Primer Type | Sequence |
| --- | --- | --- |
| CCR7 | F | 5’-AGCAACTCAACATCGCCTACG-3’ |
|  | R | 5’-CAAGAAAGGGTTGACGCAGC-3’ |
| I11B | F | 5’-GGGGAGATCATCGGGACAACTC-3’ |
|  | R | 5’-AGAATGGCCGAGTTCATGAGCT-3’ |
| FOSB | F | 5’-CGACCCCTCCAGGAAGAAC-3’ |
|  | R | 5’-TCCTGGTTGTGCCGTTTG-3’ |
| MMP9 | F | 5’-GGCTGTGACTATGGTTACAC-3’ |
|  | R | 5’-CCTCGCGGCAAGTCTTCAG-3’ |
| α-SMA | F | CGTACAACTGGTATTGTGCTG |
|  | R | ACAGAGTACTTGCGTTCTGG |
| SM22α | F | AGTGGAGTGGATTGTAATGCAG |
|  | R | TGTTCCATCTGCTTGAAGACC |
| OPN | F | ATGACGATGATGATGACGATGG |
|  | R | AACTTGTGGCTCTGATGTTCC |
| GAPDH | F | 5’-TGAAGGTCGGAGTCAACGGATTTGGT-3’ |
|  | R | 5’-CATGTAGGCCATGAGGTCCACCAC-3’ |

F, Forward; R, Reverse.

| Rank | Methods | | | | |
| --- | --- | --- | --- | --- | --- |
|  | MNC | MCC | EPC | Degree | Closeness |
| 1 | PTPRC | PTPRC | PTPRC | PTPRC | PTPRC |
| 2 | LCP2 | LCP2 | LCP2 | LCP2 | LCK |
| 3 | RAC2 | BTK | LCK | RAC2 | RAC2 |
| 4 | LCK | RAC2 | RAC2 | LCK | LCP2 |
| 5 | IL1B | HCK | CD48 | IL1B | WAS |
| 6 | WAS | WAS | IL10RA | WAS | IL1B |
| 7 | IL10RA | LCK | IKZF1 | CYBB | CYBB |
| 8 | BTK | VAV1 | BTK | IL10RA | BTK |
| 9 | CYBB | CD48 | CYBB | BTK | HCK |
| 10 | HCK | IL10RA | IL2RG | HCK | IL10RA |
| 11 | VAV1 | HCLS1 | IL1B | VAV1 | VAV1 |
| 12 | CD48 | IL2RG | CD2 | CD48 | CD48 |
| 13 | IKZF1 | PIK3CG | HCK | CCR7 | CCR2 |
| 14 | CD2 | IKZF1 | CCR2 | IKZF1 | IKZF1 |
| 15 | CCR7 | INPP5D | VAV1 | CD2 | IL2RG |
| 16 | IL2RG | CD2 | CCR7 | CCR2 | CCR7 |
| 17 | NCF4 | CD52 | WAS | IL2RG | CD2 |
| 18 | CCR2 | CCR2 | CD52 | NCF4 | NCF4 |
| 19 | IL2RB | CYBB | IL2RB | IL2RB | HCLS1 |
| 20 | CD52 | CCL4 | NCF4 | CD52 | PTK2 |
| 21 | CCL4 | IL2RB | CD27 | CCL4 | IL2RB |
| 22 | HCLS1 | NCF4 | CCL4 | HCLS1 | NCF1 |
| 23 | CD27 | CD27 | HCLS1 | CD27 | PRKCB |
| 24 | INPP5D | CCL5 | INPP5D | INPP5D | CD27 |
| 25 | CCL5 | TRAF3IP3 | PIK3CG | CCL5 | INPP5D |
| 26 | WASL | CCR7 | S1PR4 | PRKCB | ITGB7 |
| 27 | PTK2 | IL1B | CCL5 | WASL | WASL |
| 28 | PIK3CG | S1PR4 | CD22 | FLNA | CCL4 |
| 29 | NCF1 | CD68 | NCF1 | PTK2 | PIK3CG |
| 30 | PTGS2 | CD83 | PRKCB | S1PR4 | CD52 |

Table S4. The hub genes based on five plugin methods in Cytoscape.

**
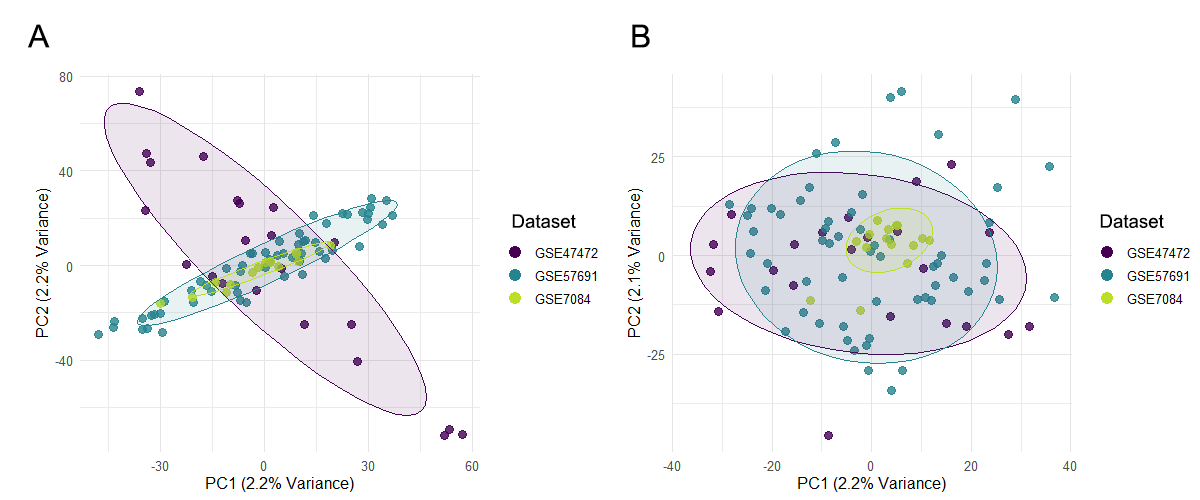
**

**Figure S1. PCA analysis before (A) and after (B) batch effect correction**

**The full uncropped blots images**

Figure S2. The full-length blots of GAPDH


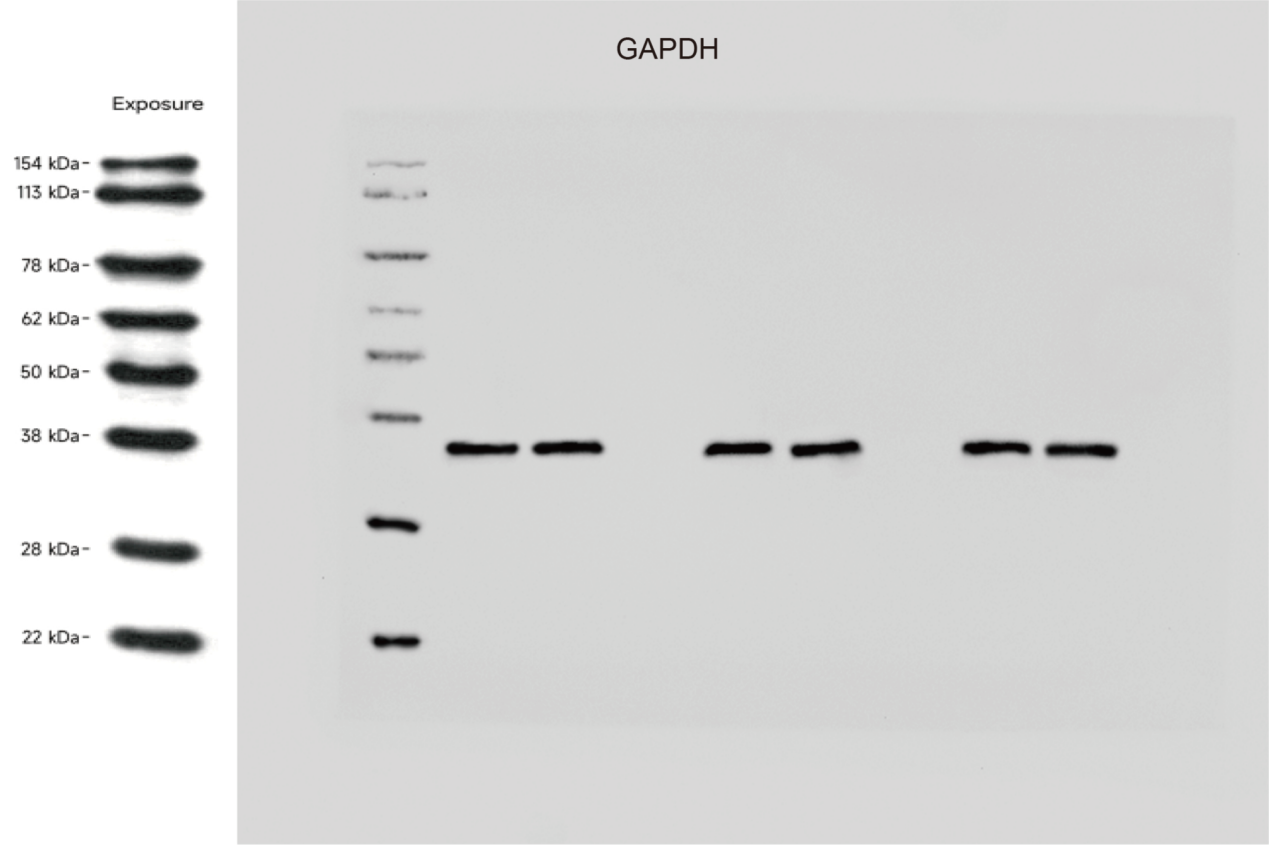


Figure S3. The full-length blots of FOSB


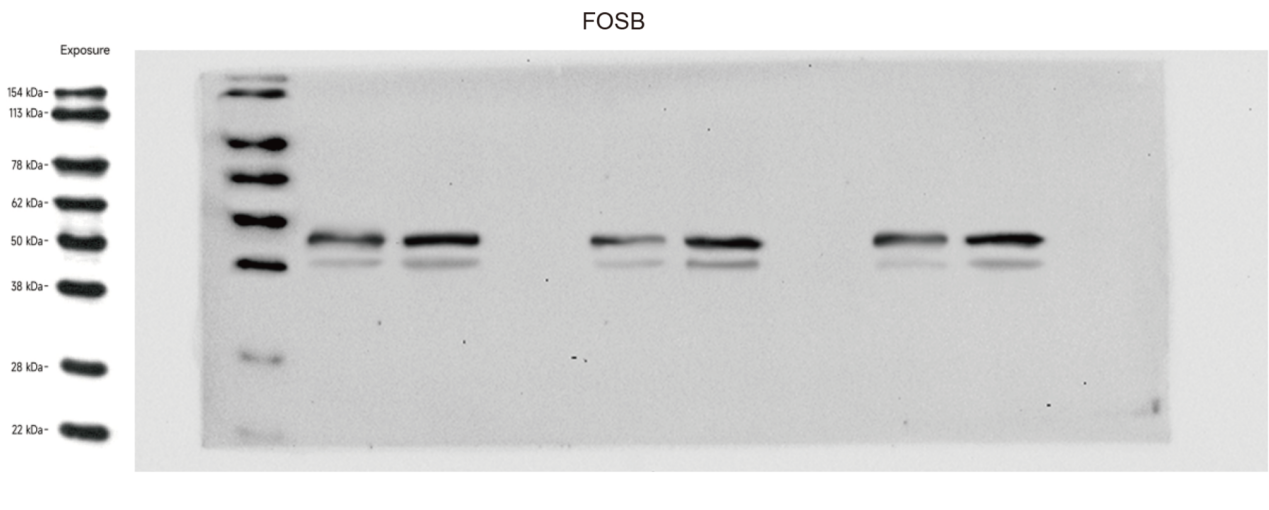


Figure S4. The full-length blots of CCR7


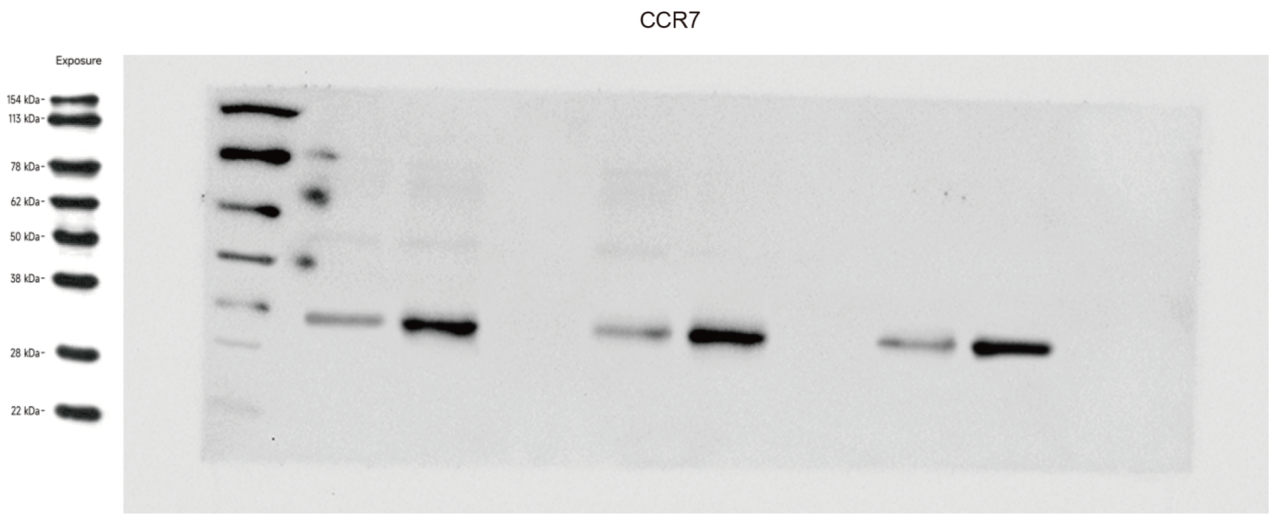


Figure S5. The full-length blots of IL1B


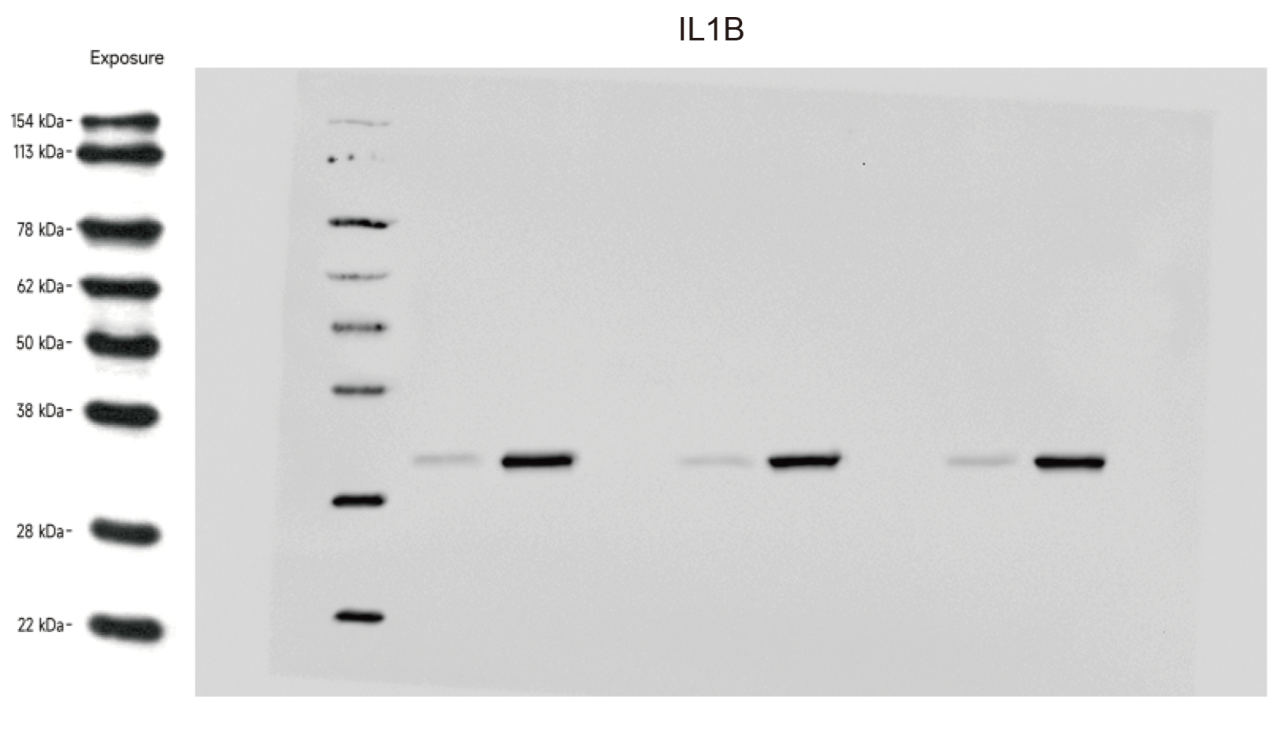


Figure S6. The full-length blots of MMP9


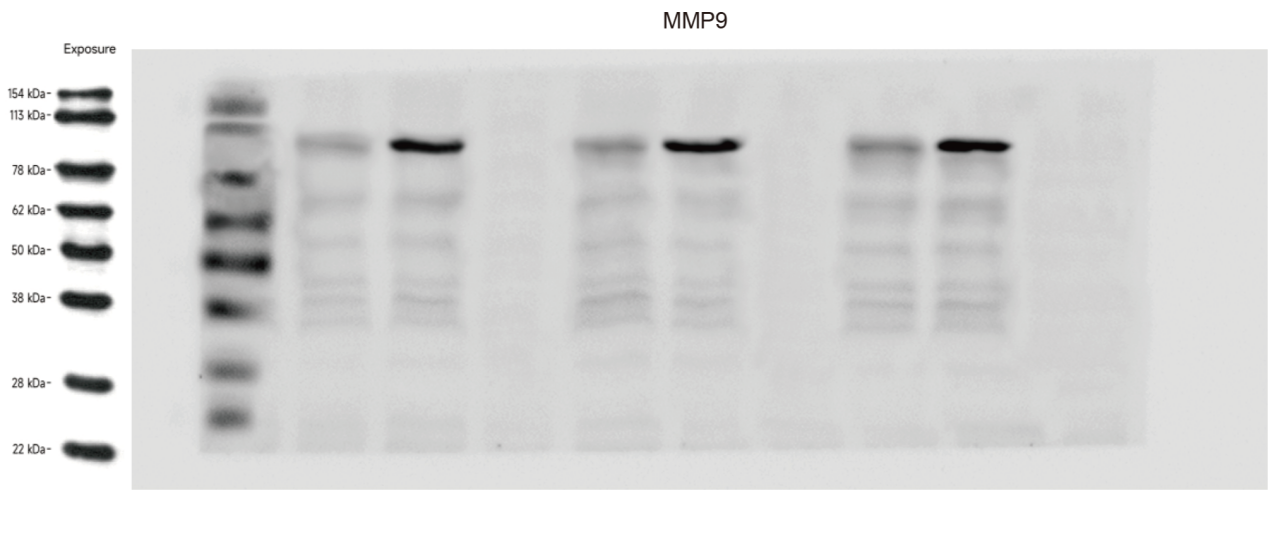

Supplement: Supplementary file 1 [file DataSheet1.docx]
